# Transcranial direct current stimulation (tDCS) in psychiatric disorders in early childhood (aged under 10 years): a systematic review

**DOI:** 10.1007/s00787-024-02635-z

**Published:** 2025-01-10

**Authors:** Hannes Brehme, Josefin Utke, Christoph Berger, Michael Kölch, Johannes Buchmann

**Affiliations:** 1 Department of Psychiatry, Neurology, Psychotherapy and Psychosomatics in Childhood and Adolescence, Rostock University Medical Center, Gehlsheimer Straße 20, 18147 Rostock, Germany; 2 German Center for Child and Adolescent Health (DZKJ), Site Greifswald/Rostock, Germany

**Keywords:** Tdcs, ASD, Systematic review, Children, Psychiatric disorders

## Abstract

Transcranial direct current stimulation (tDCS) remains experimental for many psychiatric disorders in adults. Particularly in childhood, there is limited research on the evidence for the efficacy and mechanisms of action of tDCS on the developing brain. The objective of this review is to identify published experimental studies to examine the efficacy and mechanisms of tDCS in children with psychiatric or developmental disorders in early (prepubertal) childhood (aged under 10 years). Included Studies should meet the following criteria: (1) experimental studies (no reviews, no case reports), (2) studies published in international peer-reviewed journals, (3) written in English, (4) conducted on children under 10 under years of age, (5) at enrolment with a psychiatric or developmental disorder.Eight studies were identified that fulfilled the specified criteria. All studies investigated effect on children with autism-spectrum-disorder (ASD). Anodal tDCS, mainly targeting the left dorsolateral prefrontal cortex (dlPFC), showed positive effects on the reduction of ASD symptoms. There has also been evidence that these stimulations are feasible, have good tolerability and are safe. tDCS was found to be safe and partially effective, but a long-term effect of tDCS and changes in connectivity during tDCS in autism has not been proven. Other developmental or psychiatric diseases were not investigated. This results in a lack of knowledge regarding the reactivity of the brain during the prepubertal period, which is a critical phase in the pathogenesis of neurodevelopmental disorders such as attention deficit hyperactivity disorder (ADHD), ASD, Tourette’s syndrome or dyslexia.

## Introduction

Transcranial direct current stimulation (tDCS) is a non-invasive method of brain stimulation, that modulates brain excitability and is potentially effective for various diseases [1]. As early as 1801, Giovanni Aldini achieved a mood improvement in “melancholic” patients using direct current stimulation in adults [2]. Over 200 years later tDCS remains experimental for many psychiatric disorders and is not yet approved by regulatory agencies like the FDA. To date, the pathophysiological changes in connectivity under tDCS has mainly been investigated in adults. Focus of psychiatric research were several disorders as depression, anxiety and obsessive-compulsive disorder [3]. Neurological studies can be found on Alzheimer’s disease, Parkinson’s disease, pain, epilepsy, and rehabilitation after stroke [4–6]. Research in children has so far examined the effects of tDCS on attention deficit hyperactivity disorder (ADHD), autism-spectrum-disorder (ASD), dyslexia, epilepsy, infantile cerebral palsy and motor learning [7–10].

Non-invasive neuromodulation methods as tDCS are safe treatments in adults in various psychiatric disorders (major depressive disorder, schizophrenia, anxiety and obsessive-compulsive disorder) [11]. Bikson et al. [12] and Antal et al. [13] published reviews about safety and also about ethical, legal regulatory aspects. Both declaim that the use of conventional tDCS protocols in human trials (≤ 40 min, ≤ 4 mA, ≤ 7.2 C) has not produced any reports of a serious adverse effects or irreversible injury. tDCS is straightforward, well-tolerated, and carries minimal adverse effects, such as mild erythema and pruritus, and a reduced probability of developing headaches, when compared to repetitive transcranial magnetic stimulation (rTMS) (11.8% in tDCS and 23% in rTMS) [14]. It has already been shown for ADHD in adulthood that use at home is possible and safe [15]. This indicates that this method may be applicable in younger age groups. To illustrate the variety of “young age”, the review by Gallop et al. [16] included participants up to the age of 25 years due to the limited data in the preschool age group. Methodological differences may exist between adults, adolescents and children: In adulthood there are well-known principles for strategic positioning of the electrodes [17], about changes in excitability under anodal or cathodal electrode [18, 19] and even long lasting effects over NMDA related cortical long-term potentiation and depression [20–23]. The data concerning the effects of tDCS on the developing brain are currently lacking; however, the safety of its application in younger age groups has been demonstrated [24]. In some MRI studies current flow was modelling the resulting electric field during tDCS [25], which highlighted the necessity for adaptations to the stimulation parameters in childhood. Other earlier studies noticed, that age-specific influences of tDCS on cortical excitability of the primary motor cortex [26]. But nevertheless, the extent of influence of tDCS on other regions and changes in connectivity for example by stimulation of left dorsolateral prefrontal cortex (DLPFC) is not fully understood. In child and adolescent psychiatry DLPFC is one target region for example in attention deficit and hyperactivity disorder (ADHD) [27]. The effect of neuromodulation on the DLPFC is known in adults [28] but little in developing brain. Due to this aspect, the present review has concentrated on the prepubertal age, when the development of the prefrontal cortex and its connections is ongoing and therefore potentially more vulnerable. In contrast to neurological diseases, where the focus of stimulation is more clearly defined, for example, by MRI-visible changes, in psychiatric diseases and developmental disorders, the selection of the most appropriate stimulation area should be based on an understanding of the connections and brain development involved. As early as 2014, Davis wrote that there has been limited research into the effectiveness due to: (1) unknown effect of the stimulation (2) unknown side effects of the stimulation (3) lack of clear dosage guidelines, (4) lack of transferable studies from adults to children [29].

### Aim of the study

In this systematic review we want to investigated the impact of tDCS on children with developmental and psychiatric diseases in early (prepubertal) childhood (younger than 10 years) as a period of increased prevalence of various neurodevelopmental disorders, including ADHD, ASD or dyslexia.

## Methods

### Search strategy

**Fig. 1:**
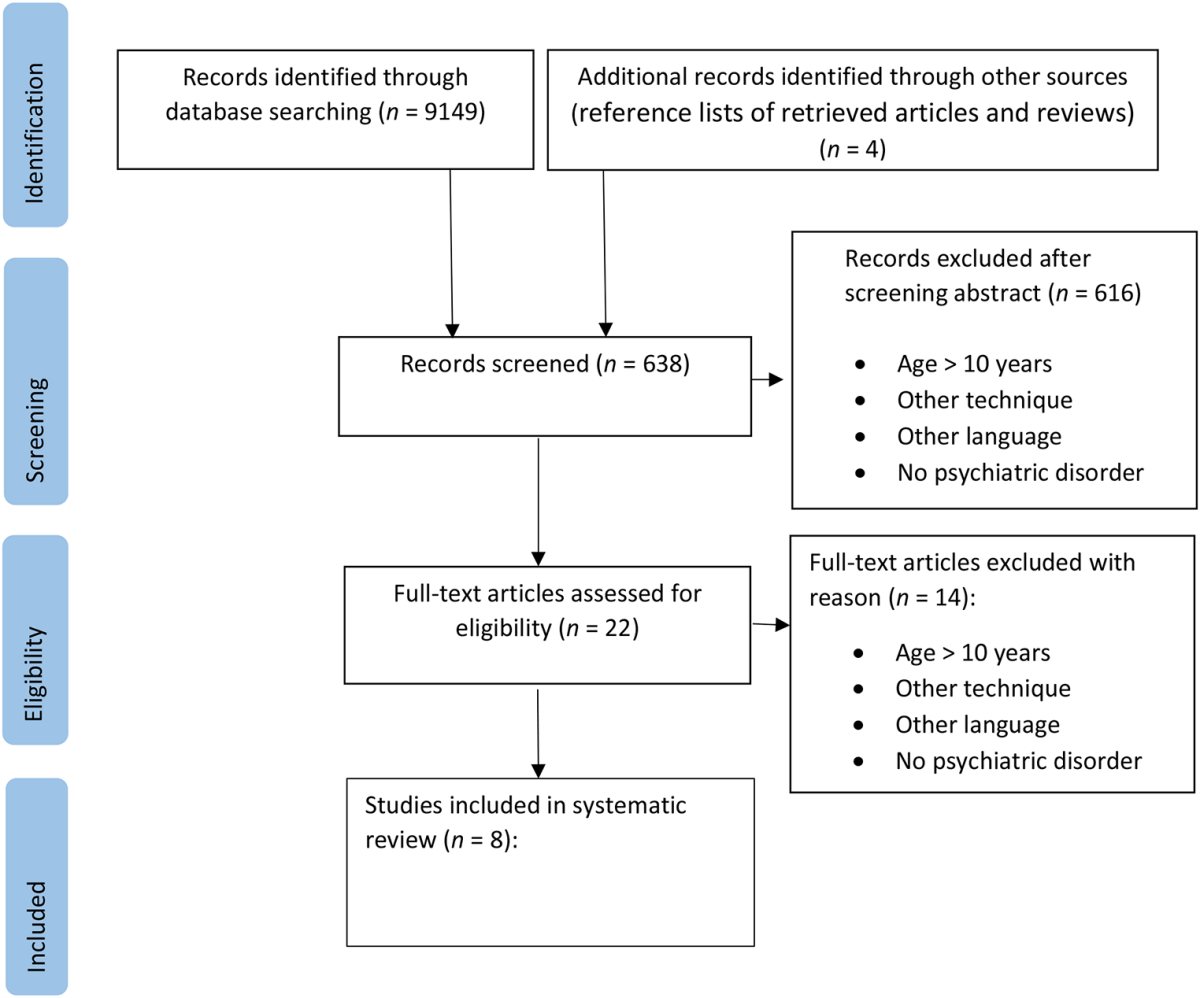
PRISMA flow diagram of included studies

We searched Web of Knowledge, Scopus, PubMed, psyarxiv, PsycINFO using the following keywords: “transcranial electric stimulation,” “transcranial direct current stimulation,” “tDCS,” each in combination with “hyperkinetic disorder,” “attention-deficit/hyperactivity disorder,” “ADHD”, “autism”, “dyslexia”, “tic”, “Tourette”, “anxiety”, depression”, “developmental disorder”, “psychiatric diseases” or “enuresis”. The authors (H.B. and J.U.) conducted a preliminary screening of titles and abstracts to identify potentially eligible studies and exclude duplicates. The full texts of the selected studies were retrieved and independently assessed by each author. We also hand-searched the reference lists of retrieved articles and reviews. Search was concluded in March 2024. The PRISMA flow diagram is displayed in Fig. 1.

### Inclusion criteria

Studies should meet the following criteria: (1) experimental studies (no reviews, no case reports), (2) studies published in international peer-reviewed journals, (3) written in English, (4) conducted on children under 10 under years of age, (5) at enrolment with a developmental or psychiatric disorder.

## Results

**Fig. 2:**
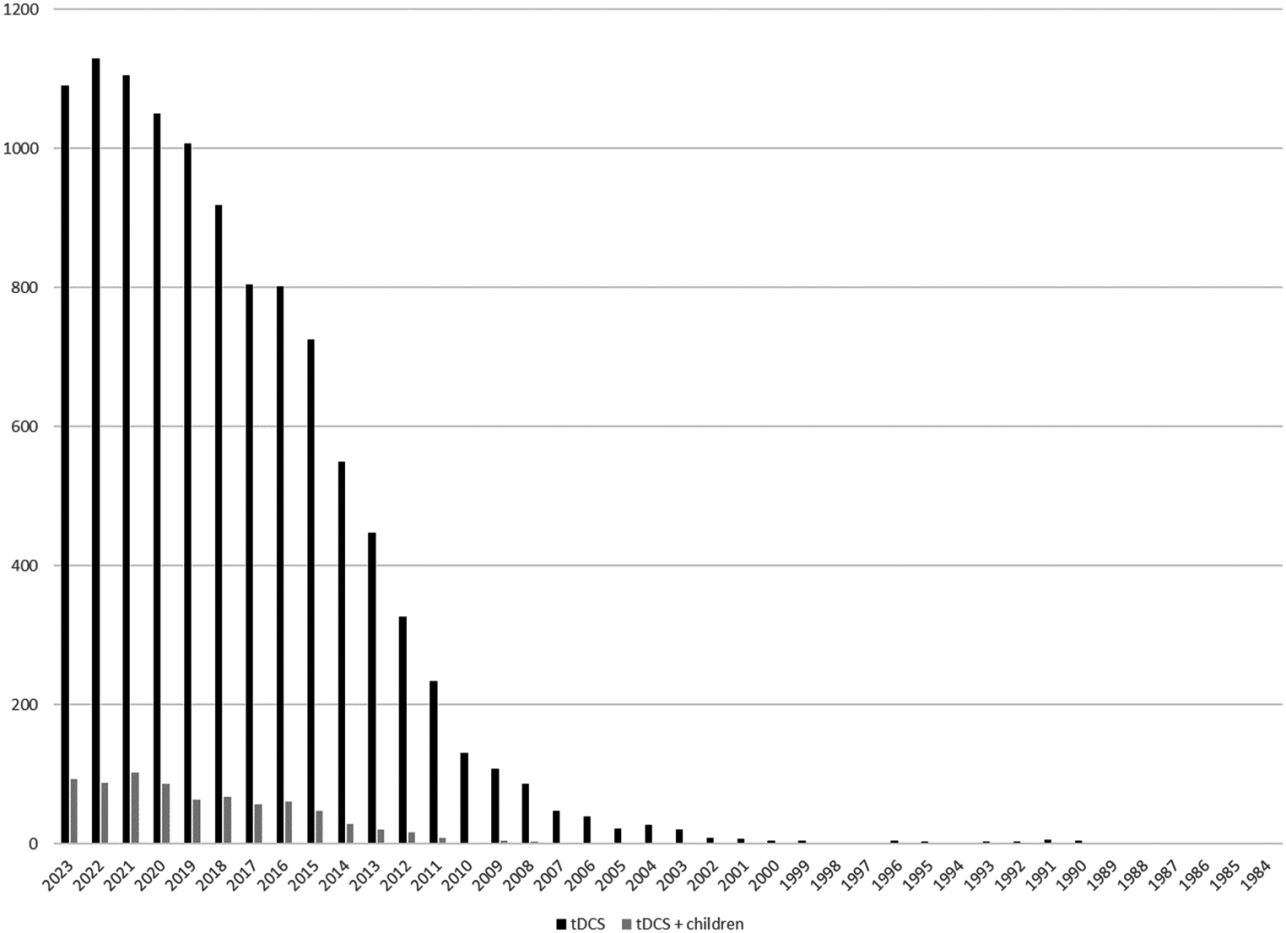
Results for searching “tDCS” (black) and “tDCS and children” (grey) during the years

In order to illustrate the current focus of research, we have plotted the number of search results from Pubmed for “tDCS” and “tDCS and children” per year in Fig. 2. Over the last few decades, there have been over 10,000 results on tDCS, but only 727 that also had the keyword children. Furthermore, only eight eligible trials included children under 10 with developmental or psychiatric disorders. All of them investigated tDCS in children with ASD. No trials were found for other psychiatric or developmental disorders. This means that the following results focus on safety, tolerability and effects in children with ASD. The eight included studies involved a total of 1,080 sessions conducted with 234 children (aged between two and eight years) diagnosed with ASD. Table 1 summarizes the stimulation protocols, sample size, and major findings of these studies.

**Table 1:** Table with studies included only children under 10 years with psychiatric diseases

Authors	Design	*N* (sex)	Mean age (range)	Disease	Stimulation method	Other treatments	Target parameters	Treatment	Results
Amatachaya et al. [30]	RCT double-blind (sham controlled)	20 (males)	6.4 (5-8)	ASD	Anode: left DLPFC (F3) Cathode: right shoulder Electrode size: 7 × 5 cm Intensity: 1 mA	Medication (5 patients) All patients: Behavioral therapy (e.g. animal assisted therapy; speech therapy)	CARS ATEC CGAS CGI-I	8 weeks 10 × 20 min (daily) (5 active + 5 sham)	CARS score were reduced after seven days of active tDCS statistical change in total ATEC score observed in the active tDCS treatment CGAS showed statistical increase in the active tDCS treatment 9 from 20 of active tDCS were rated as “much improved” in CGI
Amatachaya et al. [31]	RCT double-blind (sham controlled)	20 (males)	6.4 (5-8)	ASD	Anode: left DLPFC (F3) Cathode: right shoulder Electrode size: 7 × 5 cm Intensity: 1 mA	Medication (5 patients) All patients: Behavioral therapy (e.g. animal assisted therapy; speech therapy)	ATEC EEG (peak alpha frequency)	3 weeks 20 min (each session)	Improvements in two domains of ATEC (social and health/behavior domains) following active tDCS PAF increased and an increase in PAF was significantly associated with improvements in domains of ATEC impacted by tDCS
Kang et al. [37]	RCT wait-list control trial	26 (11 males; 2 females)	(4-8)	ASD	Anode: left DLPFC (F3) Cathode: right supraorbital (Fp2) Electrode size: 7 × 4.5 cm Intensity: 1 mA	No information	EEG (maximum entropy ratio method)	3 weeks 10 × 20 min (every 2 days)	MER value significantly increased after tDCS treatment (active tDCS)
Auvichayapat et al. [35]	Open-label, single-arm study	10 (male)	6.6 (5-8)	ASD	Anode: left DLPFC Cathode: right shoulder Electrode size: 7 × 5 cm Intensity: 1 mA	Medication (3 patients) All patients got non-medication treatment	ATEC CARS Secondary outcome: levels of brain metabolites	2 weeks 5 × 20 min (daily)	Significant decrease in ATEC social subscale scores, significant increases in N-acetylaspartate (NAA)/creatine (Cr) and myoinositol (mI)/Cr-concentrations, and a decrease in choline (Cho)/Cr concentrations in the left DLPFC and locus coeruleus after tDCS treatment
Qiu et al. [36]	RCT single- blind (sham controlled)	40 (10 females; 30 males)	(2-6)	ASD	Anode: left DLPFC (F3) Cathode: right shoulder Electrode size: 5 × 5 cm Intensity: 1 mA	No information	CARS ABC RBS-R Secondary outcomes: sleep condition, as measured by CSHQ	3 weeks 5 × 20 min (per week)	Real tDCS, but not sham tDCS, significantly reduce the scores of CARS, Children’s Sleep Habits Questionnaire (CSHQ), and general impressions in CARS
Auvichayapat et al. [33]	Double-blind, multi- arm RCT	36 (males)	6.2 (3-7)	ASD	Anode: left DLPFC Cathode: right shoulder Electrode size: no information Intensity: 1 mA	Medication (12 patients)	CARS ATEC	12 months 5 or 20 sessions	Significantly reductions in autism severity in 5-tDCS treatment and 20-tDCS treatment
	Double-blind, multi- arm RCT	52 (44 males; 8 females)	(3-6)	ASD	Anode: left DLPFC Cathode: right supraorbital (Fp2) Electrode size: 7 × 4.5 cm Intensity: of 1 mA	No information	ABC EEG microstates	5 weeks 10 × 20 min twice a week	tDCS displayed significant differences in EEG microstate and ABC scale between pre- and post-tDCS in the experimental group
	Double-blind, multi- arm RCT	30 (no information)	(3-8)	ASD	Anode: primary motor cortex Cathode: right deltoid muscle Anode: cerebellum Cathode: central supraorbital region Electrode size: 5 × 7 cm2 Intensity: 1 mA	Information about the medications will be collected	Autism Diagnostic Observation Schedule (ADOS) (CARS-BR) Movement Assessment Battery for Children-2 (MABC-2) Secondary outcomes: Functional balance: Pediatric Balance Scale Functional mobility: The Timed Up and Go (TUG) test	2 weeks 5 × 20 min (5 days per week)	Recruitment of the participants began in January 2023 and end in December 2023

### Quality of studies

Out of the eight studies, two had a double-blind randomized controlled design [30, 31] and three had a double-blind, multi-arm randomized controlled design [32–34]. One study [35] was an open, single-arm study, so there was no control group. The study by Qui et al. [36] used a single-blind design. Kang et al. [37] had a randomized controlled waitlist design. The revised Cochrane risk of bias tool [38] was used to assess the risk of bias, and it was found that only minimal overall bias was present in the included studies. However, the limited publication of stimulation protocols constrained the comparability of the results. In addition to the interventions, specific medications and non-medical treatments were reported in only five out of eight trials [30–33, 35].This leads to heterogeneity in the possible assessment of effectiveness.

### Safety and tolerability

The over 1080 sessions included in the studies were well tolerated, with no serious adverse events reported. In particular, tDCS has been demonstrated to be a highly acceptable intervention, with all enrolled participants successfully completing their respective protocols. In two studies [30, 35], no adverse events were reported in the active or sham group or observed by the investigators. One study observed adverse events, but did not report them in the results [34] and another study [37] does not provide any information on adverse events.

Three studies reported, that tDCS is only associated with minor side effects. Skin irritation was the most common side effect, disappearing within a few days. One study reported transient erythematous rash in three children who received active tDCS [31]. In one study [33], the caregivers of one child reported insomnia and irritation on the second night after tDCS and another in the tDCS group was reported to have been irritable on the second and third day after tDCS. In another [36] study, a parent reported hyperactivity after one week of real tDCS treatment for one child. The symptoms disappeared within a week after tDCS treatment.

### tDCS effects in ASD

In all studies, the main aim was to investigate the potential of tDCS to reduce the intensity of ASD related symptoms. As can be seen in Table 1, all studies chose 1 mA as the stimulation intensity. All included studies investigated the efficacy of tDCS and suggest that anodal tDCS may be a useful clinical tool in ASD. The first identified study was conducted by Amatachaya et al. [30], in which 20 patients received 5 consecutive days a sham or active tDCS.

Assessments of ASD symptom severity and overall functioning were measured by the CARS, ATEC and CGAS. These were administered before treatment and 7 days after treatment. The CGI-I was also administered at 7 days post-treatment to assess overall improvement in autism. Active tDCS resulted in symptom alleviation, while in the sham condition (baseline vs. 7 days after treatment), there were no changes in symptoms. A statistically significant improvement in ASD symptom severity was observed according to the CARS and the ATEC global score (overall improvement). Additionally, there were improvements in health, behavioral problems, sociability, and sensory/cognitive awareness. The CGAS score was also increased at 7 days post-treatment.

Another study [36] with 40 children (20 active tDCS vs. 20 sham tDCS) could show that active tDCS, but not sham tDCS lead to a significant reduction in the scores of CARS and significant improvement in sleep (CSHQ), but revealed no statistically significant difference in pre- and post-treatment changes for ABC, CARS and RSB scores between the real and sham tDCS groups. Overall, result suggest that active tDCS relieving some symptoms in children with ASD.

More recently, Auvichayapat et al. [33] comparing three treatment conditions (a) 5 sessions of anodal tDCS, (b) 20 sessions of anodal tDCS or (c) sessions of sham tDCS in 36 children with ASD and reported, compared to the control group, the 5-session and 20- session with tDCS showed a significant reduction in autism severity and related symptoms at days 5 and 14, and months 6 and 12. But there were no significant differences in the outcome between the 5-and 20-tDCS group at any time.

Three included studies investigated the improvement of symptoms associated with ASD through tDCS and the changes in the electroencephalogram (EEG) before and after tDCS. The first study [31], assed the effects of anodal tDCS on peak alpha frequency (PAF) related to ATEC scores. Previous studies indicating a reduced alpha activity in children with ASD, therefore the authors sought to investigate whether tDCS-induced PAF increases would be associated with symptom alleviation in ASD. A total of 20 male children with autism were randomly assigned in a crossover design to receive either active or sham tDCS. The results demonstrate that active tDCS induces significant pre-to-postsession improvements in two subscales of ATEC (social and health/behavior domains) relative to sham treatment. Furthermore, PAF was found to have increased significantly at the stimulation site. Additionally, an increase in PAF was found to be significantly associated with improvements in the two domains of ATEC that were impacted by tDCS.

Another study [37] also measured changes in the complexity of EEG series and used the MER method. This indicating an imbalance between excitatory and inhibitory in the neuronal network which may plays a role in the pathogenesis of ASD. The aim of this study was to investigate the effects of tDCS over DLPFC on EEG activity in children with ASD and that tDCS would be an effective method for altering the excitatory and inhibitory imbalance. A total of 13 children with ASD received 10 active tDCS treatments over the DLPF every two days. Another 13 children with ASD, who were awaiting therapy constituted the control group. The results shows, that the MER value was higher when comparing post-tDCS to pre-tDCS for experimental group and almost remained unchanged for controls, which means that the EEG complexity increased after one session.

The third study [34], which analyzed differences in EEG microstates and examined the ABC scores before and after tDCS in three groups: (a) tDCS treatment, (b) sham tDCS, and (c) a typical development group (TD). In the experimental group, the scores of EEG microstates before tDCS differed from those after tDCS. Conversely, In the control group, there were no significant differences in the EEG microstate or ABC scores before and after sham stimulation.

## Discussion

In this review, we examined the effectiveness, safety, and tolerability of tDCS in developing brain (under the age of 10) with developmental or psychiatric disorders. All studies included in our review focused on children with ASD, a condition characterized by impaired communication and language, social interaction deficits, and restricted or repetitive behavior [39]. Our findings indicate that tDCS lead to improvements in at least one of the outcome measures, supporting its potential to improve symptoms and reduce severity in ASD. The stimulation of the left DLPFC reports promising effects in the reduction of behavioral problems in ASD and the results encourage further research into tDCS as a potential treatment option for children with ASD.

Most studies have targeted the left DLPFC [30, 31, 33–37] as it is a central region for executive function and research suggests that the DLPFC and its connectivity are involved in the pathophysiology of ASD [40]. Only one study [32] has been conducted to assess the impact of targeting the primary motor cortex and cerebellum, both of which are involved in the pathophysiology of ASD. The included studies differed in their interventions and in the duration of tDCS, which may explain the variability of the results and the different effects. For example, in the study by Amatachaya et al. [30], children received 10 × 20 min of active tDCS over five days. In contrast, participants in the study by Kang et al. [37] received 10 × 20 min of tDCS every two days. Auvichayapat et al. [35] only stimulated the DLPFC for 5 × 20 min. Interestingly, the study by Auvichayapat et al. [33] showed that there was no difference in outcome whether 5-session or 20-sessions tDCS intervention was used. But there is a limitation in long-term observation. Only one study [33] measured the effects at a long-term follow-up of 12 months after the tDCS session. This indicates, that long-lasting effects of tDCS in ASD has not been proven and shows that more research on long-term effects is needed.

With regard to safety and tolerability, three studies [31, 33, 36] reported very minor side effects. tDCS appears to be safe and tolerable, with only mild discomfort in a minority of children and no serious adverse events. This is consistent with reviews and meta-analysis focusing on safety [41, 42].

Additionally, the mechanisms of action of tDCS are not yet fully understood. In patients with ASD, altered functional connectivity between frontal and other cortical areas has been described as a possible mechanism of the pathophysiology of ASD [43]. Furthermore, an imbalance between excitation and inhibition in the synaptic transmission and neural circuits have been demonstrated [44]. In this regard, the results of Kang et al. [37], who measured tDCS-induced changes in EEG complexity in children with ASD, showed that tDCS over the DLPFC increased cortical excitability and improved the balance between excitation and inhibition of neurons. Amatachaya et al. [31] have shown that tDCS simulation leads to an increase in alpha frequency, reflecting an increase in synaptic connectivity, and that this tDCS-induced increase in alpha frequency was associated with symptom improvement in ASD in those who received tDCS. However, three [31, 34, 37] of the eight studies, which reported EEG changes, these were measured only before and after tDCS, not during tDCS. But little is known about changes in connectivity during tDCS. Especially in still-developing brains, where use-dependent plasticity changes are strongest [45], knowledge of connectivity changes during tDCS could provide insights into structural connections and the mechanisms of action of the brain. This underlines the need to improve the understanding of the changes of connectivity under tDCS in developing brain are needed.

### Limitations

While there is evidence that certain interventions may be effective in reducing behavioural symptoms associated with ASD, it is important to note that the studies included in this review have several limitations. Consequently, the results should be interpreted with caution. First, the heterogeneity of the included studies limit the interpretation and comparability of the results. In order to investigate the clinical significance of tDCS intervention in children with ASD, it is necessary to conduct randomized controlled trials. Of the eight studies included in the review, four were randomized, double-blind, sham-controlled trials [30–33]. A further limitation is the sample size and gender distribution in some included studies. Some studies used a limited number of samples. In the studies by Amatachaya et al. [30, 31], only 20 children were examined and they also used the same sample in both studies. Another study included only 10 participants [35]. In addition, four of the eight studies [30, 31, 33, 35] included only male children. It is not possible to determine whether the results can be generalised to females with ASD.

In addition, it is important to note that three of eight studies reported on patients who were taking medications to treat their symptoms. For instance, five out of twenty participants in the studies by Amatachaya et al. [30, 31] regularly took medication (risperidone) to treat their symptoms, while all others did not take any medication. In the study by Auvichayapat et al. [35] three out of ten children reported that they received medication. Another study [33] reported that 12 of 36 participants received medication. The information about the medication use is a crucial point, as there may be interactions between tDCS and medication. None of the studies mentioned above controlled for medication use and did not examine the potential moderating effects of medication on tDCS treatment. The review by McLaren et al. [46] demonstrated that several classes of drugs, including those that affect different neurotransmitter systems, may influence the effect of tDCS on tissue excitability. These findings highlight the importance of documenting participants’ medication use. Future studies could investigate the moderating effects of medication intake on tDCS treatment. At a minimum, take medication use into account in data analysis.

## Conclusion

To date, research into the use of tDCS for the treatment of psychiatric and developmental disorders in children has largely focused on ASD, with stimulation primarily targeting the left DLPFC. The feasibility, tolerability and safety of these stimulations have been demonstrated. As all studies used the same stimulation intensity (1 mA), they are comparable, but a dose-response relationship is not possible at this vulnerable stage of development. However, long-term observation was only conducted in one study, and thus no definitive conclusions can be drawn regarding the long-term effects. Currently, there is limited knowledge of changes in connectivity during tDCS in the developing brain. In contrast to neurological diseases (mainly infantile cerebral palsy (review [47]), where the focus of stimulation is more clearly defined, changes in developmental and psychiatric disorders in early childhood are poorly investigated. As in the studies reviewed here, the DLPFC appears to be a promising target for tDCS in several neuropsychiatric disorders [48]. While there have been studies of tDCS in other developmental disorders, such as ADHD and dyslexia [10], these have only been conducted in adolescents and poorly in younger children. This leads to a lack of understanding how the brain responds during the vulnerable period. For neurodevelopmental disorders such as ADHD, ASD, Tourette’s syndrome or dyslexia, this is a crucial period. As changes in plasticity start in prepubertal age, there is a need to understand the varying status of connectivity in developing and developed brains [49]. As noted above, there are age-specific influences of tDCS [26], but the extent of these influences on neuromodulation remains largely unclear [50].

## Data Availability

No datasets were generated or analysed during the current study.
